# Immune Checkpoint Inhibitor-Associated Myocarditis: Risk, Diagnosis, and Clinical Impact

**DOI:** 10.3390/jcm15020814

**Published:** 2026-01-19

**Authors:** Alfredo Mauriello, Adriana Correra, Anna Chiara Maratea, Valeria Cetoretta, Giovanni Benfari, Federica Ilardi, Rosangela Cocchia, Matteo Lisi, Alessandro Malagoli, Giulia Elena Mandoli, Maria Concetta Pastore, Simona Sperlongano, Vincenzo Russo, Matteo Cameli, Antonello D’Andrea

**Affiliations:** 1 S.C. Cardiology, Institute National Cancer, IRCCS, Foundation “G. Pascale”, Via M. Semmola 52, 80131 Naples, Italy; alfredo.mauriello93@libero.it; 2 Cardiology Department, Ospedali Riuniti University Hospital, Viale Pinto 1, 71122 Foggia, Italy; adrianacorrera@gmail.com; 3 Department of Cardiovascular Disease, ASL Napoli 1 Centro, Via Comunale del Principe, 13/a, 80145 Napoli, Italy; annachiara.maratea@gmail.com; 4 Cardiology and Arrhythmology Clinic, Marche Polytechnic University, University Hospital “Ospedali Riuniti”, Via Conca 71, 60126 Ancona, Italy; vcetoretta@gmail.com; 5 Section of Cardiology, Department of Medicine, University of Verona, P.le L.A. Scuro 10, 37100 Verona, Italy; giovanni.benfari@univr.it; 6 Department of Advanced Biomedical Sciences, Division of Cardiology, Federico II University Hospital, Via Pansini 5, 80131 Naples, Italy; federica.ilardi@unina.it (F.I.); rosangelacocchia@hotmail.com (R.C.); 7 Department of Cardiovascular Disease—AUSL Romagna, Division of Cardiology, Ospedale “S. Maria delle Croci”, Viale Randi 5, 48121 Ravenna, Italy; matteo.lisi@hotmail.it; 8 Division of Cardiology, Nephro-Cardiovascular Department, “Baggiovara” Hospital, Via P. Giardini 1355, 41100 Modena, Italy; ale.malagoli@gmail.com; 9 Department of Medical Biotechnologies, Division of Cardiology, University of Siena, Viale Bracci 16, 53100 Siena, Italy; giuliaelena.mandoli@unisi.it (G.E.M.); mariaconcetta.pastore@unisi.it (M.C.P.); matteo.cameli@unisi.it (M.C.); 10 Cardiology Unit, Department of Medical and Translational Sciences, University of Campania “Luigi Vanvitelli”, “I Policlinico” Hospital, Piazza Luigi Miraglia 2, 80100 Naples, Italy; simona.sperlongano@unicampania.it; 11 Cardiology Unit, Department of Medical and Translational Sciences, University of Campania “Luigi Vanvitelli”, “V. Monaldi” Hospital, Via Leonardo Bianchi snc, 80131 Naples, Italy; vincenzorusso@unicampania.it; 12 Cardiology and Intensive Care Unit, Department of Cardiology, “Umberto I” Hospital, Via Alfonso de Nicola 1, 84014 Nocera Inferiore, Italy

**Keywords:** myocarditis, immune checkpoint inhibitors, cardiotoxicity, cancer

## Abstract

**Background:** Immune checkpoint inhibitors (ICIs), such as anti-programmed death (PD)-1 and anti-cytotoxic T-lymphocyte-associated protein (CTLA)-4 agents, have revolutionized oncology but are associated with immune-related adverse events (irAEs). Among these, ICI-associated myocarditis (ICI-M) is a rare but life-threatening complication, with mortality rates ranging from 27% to 50%. **Objective:** This narrative review summarizes the pathogenesis, epidemiology, clinical presentation, diagnostic methods, and management strategies for ICI-induced myocarditis, specifically highlighting emerging biomarkers and immunosuppressive therapeutic approaches. **Results and Discussion:** ICI-M typically presents within the first 65 days of treatment and is significantly more frequent with combination therapies. Pathologically, it is characterized by myocyte necrosis and massive infiltration of cluster of differentiation (CD)4+ and CD8+ T-cells, often overlapping with myositis (irM/M). Diagnosis relies on a multimodal approach. Management requires immediate ICI cessation and initiation of high-dose corticosteroids as first-line therapy. For steroid-refractory cases, second-line options include mycophenolate mofetil (MMF), intravenous immunoglobulin (IVIG), and emerging therapies like abatacept and ruxolitinib. Rechallenge with ICIs after high-grade ICI-M must be approached with extreme caution by the multidisciplinary team (MDT). Emerging biomarkers and omics techniques hold promise for earlier diagnosis and risk stratification. **Conclusions:** ICI-M is a rare yet highly lethal cardiac complication demanding high clinical vigilance and timely diagnosis. Management hinges on an aggressive multidisciplinary approach, aiming to minimize toxicity while balancing oncological efficacy.

## 1. Introduction

The advancing understanding of immune mechanisms in cancer development and progression has made immunotherapies, particularly immune checkpoint inhibitors (ICIs), an integral part of oncological therapeutic strategies [1]. ICIs act by blocking immunosuppressive signals on T-cells, such as programmed death-1 (PD-1) or anti-PD-ligand (PD-L)-1, anti-lymphocyte activation gene-3 (LAG-3), or cytotoxic T-lymphocyte antigen-4 (CTLA-4), thereby reactivating T-cell cytotoxicity against tumors [2]. The introduction of these drugs has significantly improved outcomes in various cancer types and overall survival, continuously expanding their clinical indications. Evidence from randomized clinical trials demonstrates a consistent overall survival benefit: meta-analytic estimates show a global hazard ratio of approximately 0.74–0.75, with ICIs improving outcomes in nearly all solid tumors studied, including lung, head and neck, gastroesophageal, renal, hepatic, and mesothelioma cancers. Importantly, ICIs generate a distinct long-tail survival pattern, with ~10% of patients achieving prolonged “cure-like” survival [3,4]. Large real-world data from a national U.S. health system confirm that these benefits translate beyond clinical trials. In a cohort of over 27,000 patients, ICIs were associated with significant survival gains across most cancer types—including elderly and comorbid populations underrepresented in trials—and produced more than 15,800 life-years gained within five years of treatment initiation. Utilization has expanded markedly, with first-line ICI use reaching 86% in non-small cell lung cancer and 95% in melanoma by 2021 [5]. Examples of ICIs and their indications are included in Table 1 [6,7,8]. Despite their beneficial effects, ICIs are associated with immune-related adverse events (irAEs), manifestations of autoimmune side effects that can affect various organ systems, such as the skin, gastrointestinal (GI) tract, thyroid, and lungs [9,10]. irAEs are expected to occur in up to 60% of patients undergoing such treatments [9]. While most irAEs are tolerable for patients, a particularly alarming irAE is ICI-associated myocarditis (ICI-M), a rare yet potentially fatal condition. The incidence of ICI myocarditis is estimated to be around 1%, with reported ranges between 0.3% and 1.4% [11]. Despite its rarity, ICI-M is severe, leading to significant long-term cardiac complications, including arrhythmias and heart failure. The mortality rates associated with ICI-M are high, ranging from 27% to 50% in affected patients. This severe toxicity may significantly limit the clinical application of ICI-based immunotherapy [12]. ICI myocarditis is the most prevalent cardiotoxicity among all those related to ICIs. This narrative review aims to summarize the current knowledge regarding the risk, diagnosis, clinical impact, and management strategies for ICI-induced myocarditis, with a focus on emerging biomarkers and immunosuppressive therapeutic approaches.

The table does not include a dedicated column for LAG-3 inhibitors because, to date (2025), only one agent in this class has received regulatory approval. Relatlimab—approved by both the Food and Drug Administration and European Medicines Agency—is available exclusively as a fixed-dose combination with nivolumab (Opdualag^®^) for first-line treatment of unresectable or metastatic melanoma and currently represents the only clinically approved LAG-3–targeting therapy worldwide.

## 2. Materials and Methods

This review is a narrative review, aiming to provide a comprehensive synthesis and critical analysis of current knowledge, progress, and future perspectives on ICI-M. Narrative reviews are valuable for synthesizing vast and evolving areas of research, such as cardio-oncology. To identify relevant sources, a literature search was conducted, focused on the key concepts of the title and related aspects. Search modalities concentrated on the use of biomedical databases PubMed/MEDLINE and EMBASE from January 2001 to December 2025, as is standard practice in systematic and narrative reviews in the field of cardio-oncology. The search strings and key terms used for literature collection, in line with a review standard, included combinations of the following concepts (using Boolean operators such as “AND” and “OR”):

Drugs: “Immune Checkpoint Inhibitors” (ICIs), “PD-1 inhibitors,” “PD-L1 inhibitors,” “CTLA-4 inhibitors”, ‘’LAG-3 inhibitors’’.

Cardiac Condition: “Myocarditis,” “Cardiotoxicity,” “Immune-related adverse events” (irAEs).

Diagnosis and Management: “Diagnosis,” “Biomarkers,” “Cardiac magnetic resonance imaging” (CMR), “Endomyocardial biopsy” (EMB), “Immunosuppressive management,” “Treatment”.

Document selection focused on English-language articles, including reviews, retrospective and prospective studies, and editorial comments that provided information on the pathogenesis, diagnosis, and therapeutic management of ICI myocarditis. Although the process does not follow the rigorous replicability of a systematic review [13], this methodology ensures that the narrative review is evidence-based and covers the major clinical and scientific developments in the field, as highlighted by the documents included in this summary.

## 3. Mechanisms of Cardiotoxicity Regarding Immune Checkpoint Inhibitors

**Figure 1:**
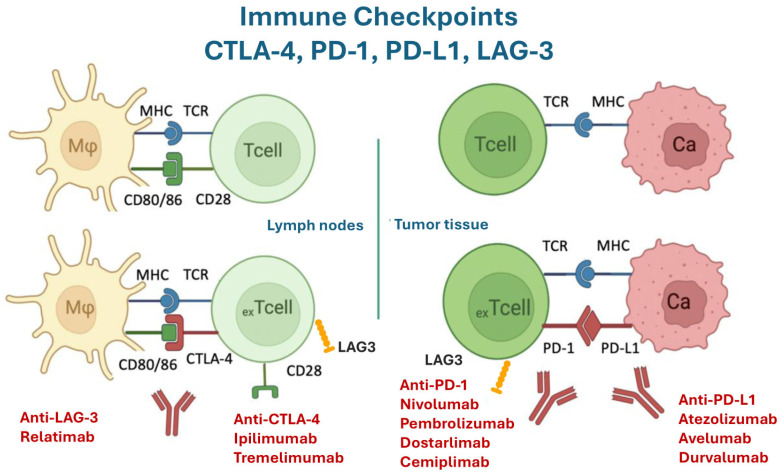
Schematic of T-cell activation and exhaustion. Naïve T-cells are primed through a two-signal process. The first signal involves the binding of the antigen to the T-cell receptor (TCR). The second signal is delivered by the interaction of activated antigen-presenting cell (APC) ligands, specifically CD80 and CD86 (also known as B7-1 and B7-2), with the CD28 receptor on the T-cell. However, when this antigenic stimulus becomes continuous (chronic stimulation), the antigen-specific T-cells enter a state of exhaustion (exTcell), rendering them functionally unresponsive. This exhaustion is mediated by the upregulation of specific negative immune checkpoints, notably CTLA-4 and PD-1. In the tumor environment, cytotoxic T-cells (CTLs) are meant to perform their effector function upon re-encountering the antigen they were primed against. However, chronic exposure to the antigen in the tumor induces PD-1 expression on the exhausted CTLs. Many cancer cells (Ca) express the ligand PD-L1. The interaction between PD-1 on the exhausted T-cell and PD-L1 on the cancer cell suppresses the T-cell’s effector function. LAG-3 plays a major role in negatively regulating T-cell function thereby providing tumors with an immune escape in the tumor microenvironment. CTLA-4: Cytotoxic T-lymphocyte-associated protein 4; LAG-3: lymphocyte activation gene-3; PD-1: Programmed Death 1; PD-L1: Programmed Death-Ligand 1.

ICI-related myocarditis is an irAE that stems from the non-specific, systemic activation of immune cells, a side effect of the ICI action of blocking immunosuppressive signals like PD-1 and CTLA-4 to reactivate T-cell cytotoxicity against tumors [14]. Although the exact mechanism remains under investigation, the pathophysiology is primarily characterized by the breakdown of peripheral immune tolerance and antigenic cross-reactivity [15]. The pathological hallmark is myocardial cell necrosis and significant infiltration of T lymphocytes, particularly cytotoxic cluster of differentiation (CD)8+ T-cells, and macrophages (CD68-positive). These clonal, autoreactive T-cells activate against muscle antigens, leading to myocyte damage [16]. Alpha-myosin has been identified as a key potential autoantigen in this condition, recognized by T-cell receptors (TCRs) and present in both the heart and skeletal muscles. This cross-reactivity explains why ICI myocarditis is often associated with other muscle toxicities, such as myositis and/or myasthenia-like syndrome [17]. Cardiac inflammation is further exacerbated by immune system dysregulation, including the expansion of pathogenic macrophages in the myocardium and the increased production of pro-inflammatory cytokines such as tumor necrosis factor (TNF)-alpha, interleukin (IL)-1 beta, and IL-6. Some studies also suggest that ICIs may increase IL-17A levels, contributing to damage. Finally, the incidence of myocarditis is higher with combination therapies and susceptibility may be influenced by variants in genes related to inflammation and cardiac structural integrity, such as titin (TTN) [18]. Figure 1 represents the mechanism of ICIs.

## 4. Risk and Epidemiology of Immune Checkpoint Inhibitors-Myocarditis

ICI myocarditis is a rare event, with a reported incidence of approximately 1%, although some studies suggest an incidence ranging from 0.3% to 1.4%. Nevertheless, the incidence of this complication is expected to increase in the coming years, both due to the dramatic expansion of ICIs’ indications and the increased diagnosis of forms that overlap with myositis and myasthenic syndromes [19,20]. Furthermore, the likelihood of cardiac toxicity from ICIs increases, and should be investigated, when other immune-related toxicities develop. The overall incidence of cardiac adverse events (CVAEs) is higher, with reported rates between 3.1% and 9.7% in some studies [21]. To date, no cases of myocarditis have been explicitly attributed to LAG-3 inhibition. In clinical trials and post-marketing data, the safety profile of relatlimab—currently the only approved LAG-3 inhibitor—does not show an increased incidence of myocarditis beyond that observed with PD-1 blockade, and no myocarditis events have been reported with other investigational LAG-3 agents [22].

Risk factors and demographics

Combination therapy: ICI myocarditis is significantly more frequent with the combined use of ICIs, particularly the combination of ipilimumab and nivolumab. One study found that the relative risk of myocarditis was 4.5 times higher in patients receiving the ipilimumab and nivolumab combination compared to nivolumab alone. The combination of nivolumab with relatlimab also showed a higher risk (1.7%) compared to nivolumab alone (0.6%) [23].

Sex and age: ICI-M appears to have a male predilection, with most cohort studies reporting a male preponderance of 67–71%. The median age of affected patients is generally older, with reported mean ages of 65–68 years. Patients aged 75 years or older have a higher risk of ICI-M, as observed in the FDA Adverse Event Reporting System (FAERS) database [24,25].

Comorbidities: The most reported risk factors include diabetes mellitus, hypertension, and smoking. Patients with a pre-existing history of autoimmune disease (AD) may be at elevated risk of CVAEs and potential cardiovascular irAEs [26].

Overlap phenotype: ICI myocarditis is commonly associated with other neuromuscular irAEs, particularly myositis and/or a myasthenia gravis-like syndrome (irM/M). Myocarditis is the most lethal complication among ICI-induced rheumatic and musculoskeletal toxicities [27].

## 5. Physiopathological Mechanism of Immune Checkpoint Inhibitor-Associated Myocarditis

Although the exact mechanism of ICI-associated myocarditis is not fully understood, emerging data have indicated several pathophysiological pathways.

### 5.1. Pathophysiological Mechanisms

There are three main potential mechanisms for ICI-M: activation of the immune system by ICIs can induce an autoimmune reaction against cardiomyocytes. One hypothesis suggests that cross-reactivity between tumor antigens and cardiac muscle antigens may trigger inflammation. Alpha-myosin has been identified as a clinically significant autoantigen in ICI-M. Alpha-myosin-specific T-cells drive immunotherapy-related myocarditis. Another potential mechanism is that ICIs may increase the level of IL-17, which has been linked to myocyte damage. Studies in murine models have also shown decreased levels of IL-10 (an anti-inflammatory cytokine) in the absence of PD-1 [15].

Preclinical studies in murine models have helped to elucidate the pathogenesis [15,28]. For instance, PD-1-deficient mouse models develop spontaneous myocarditis, and PD-1/PD-L1 deletion in Murphy Roths large (MRL) mice leads to myocarditis. Models with combined genetic knockout of Ctla4 and Pdcd1 showed that macrophages and CD8+ T-cells drive the myocarditis [29].

### 5.2. Pathological Features

The gold standard for the definitive diagnosis of myocarditis is EMB for detailed histopathological evaluation of myocardial tissue [30]. However, the clinical utility of EMB is limited due to its low sensitivity, risk, and the possibility of false negatives resulting from sampling error, given the heterogeneous distribution of cardiac inflammation. To reduce sampling error, obtaining multiple tissue samples is recommended [31].

The hallmark pathological feature of ICI-M is myocardial cell necrosis and significant infiltration of CD4+ and CD8+ T-cells, a picture that can resemble acute rejection after heart transplantation. Lymphocytic infiltration also includes CD68-positive macrophages [32,33]. Infiltration is primarily confined to the heart and skeletal muscle, while adjacent tissues, such as smooth muscle, are usually spared. Some cases may also show infiltration of eosinophils, giant cells, and neutrophils [34].

ICI-M can manifest in high-grade or low-grade forms [34]. Classification depends on the density of inflammatory infiltration, with high-grade associated with more significant myocyte necrosis and worse clinical outcomes. Table 2 classifies the grading of ICI-M sec. common terminology criteria for adverse events (CTCAE), version 5.0 [35].

## 6. Diagnosis, Clinical Presentation, and Clinical Impact

### 6.1. Clinical Presentation and Overlap Phenotypes

ICI-induced myocarditis manifests with a variable timeframe. Median time of onset appears to be 27–34 days after starting ICIs (inter-quartile range: 18 to 75 days), but it can occur early, or even late, up to two years after initiating ICI therapy, since immune-related adverse effects are idiosyncratic and not time- or dose-dependent [15,36]. This delayed heart failure could reflect remodeling in the setting of smoldering inflammation and has essential implications in long-term monitoring, as the mortality rate may be similarly high in early- and late-onset cases [25].

Severe ICI-M begins with electrical or hemodynamic instability and is clearly evident. However, there are cases in which myocardial involvement can be progressive with non-specific clinical manifestations. More than 50% of cases present preserved left ventricular ejection fraction (LVEF) at diagnosis [37].The most common symptoms of myocarditis and pericarditis is chest pain. Other common presentations include dyspnea and/or arrhythmias of different degrees and, less frequently, syncope [38,39]. ICI myocarditis often involve the conduction system, as the EKG is abnormal in more than 89% of cases, but the alterations are not predictive of subsequent clinical evolution [25,40]. Arrhythmias can range from tachyarrhythmias to bradyarrhythmias, such as complete heart block (CHB) [40,41]. Conduction disorders leading to heart failure (HF) with reduced ejection fraction (EF), occurring in about half of the patients [25,42]. A critical aspect is the frequent co-occurrence of ICI-M with other irAEs, particularly peripheral myositis and myasthenia gravis-like syndrome [43]. This overlap phenotype (irMyocarditis/Myopathy, irM/M) is now recognized as the most serious. Patients with concomitant irM/M have a higher prevalence of clinically significant arrhythmias compared to patients with isolated myocarditis, who have a higher number of acute decompensated HF events [37,44]. However, the severity of these conditions may be due to late diagnosis, as until now systematic investigation of myocardial damage in myositis conditions was not recommended. For this reason the association between myositis, myasthenia gravis, and myocarditis makes high clinical vigilance essential, even with non-specific symptoms.

### 6.2. Diagnostic Criteria and Imaging

Timely and accurate diagnosis of ICI-M is necessary to guide treatment and improve prognosis. Diagnosis relies on a comprehensive consideration of clinical symptoms and various test results [45,46].

Cardiac biomarkers: cardiac troponin (cTn) is a sensitive and specific marker of myocardial injury, with elevated levels inevitable in the presence of injury. Studies have found that 94% of ICI-M cases had elevated troponin levels [47]. Elevation of cTn or a significant change from baseline is a key criterion in clinical diagnosis. The increased cTn level can also indicate the severity of cardiac damage [48]. Recently, Lehmann et al. investigated the diagnostic and prognostic utility of cardiomuscular biomarkers in immune checkpoint inhibitor (ICI)-related myocarditis. In a prospective Franco-German cohort of 60 patients, cardiac troponin T (cTnT) proved more sensitive than cardiac troponin I (cTnI) and creatine kinase (CK) for both diagnosis and disease monitoring. A cTnT value ≥ 32× the upper reference limit within the first 72 h of admission was associated with an 11-fold higher risk of major cardiotoxic events (MACE) within 90 days. cTnT also remained elevated longer than cTnI and CK, reflecting the overall muscle injury burden and the frequent coexistence of immune-mediated myositis. The study therefore identifies cTnT as the preferred biomarker for diagnosis, risk stratification, and follow-up in ICI-related myocarditis, suggesting that current diagnostic recommendations should be reconsidered [37].

Electrocardiogram (ECG): ECG is part of the first-line diagnostic evaluation. Changes have been described in ICI-M, including sinus tachycardia, prolonged QRS and QT, reduced QRS voltages, and repolarization abnormalities [49].

Echocardiography: To date, no diagnostic echocardiographic pattern of immune-related myocarditis has been described. In this context, transthoracic echocardiography should be used as a first-line test to rule out alternative diagnoses and perform hemodynamic triage. When the diagnostic suspicion is strong, it is also a very powerful tool for prognostic stratification when using strain techniques (global longitudinal strain, GLS/global circumferential strain, GCS/global radial strain, GRS), integrated with troponin and CMR. Unfortunately, the test is not very sensitive for early screening.

When ICI myocarditis is suspected, echocardiography plays a central role in the initial assessment. It provides a comprehensive evaluation of LV and RV systolic and diastolic function (including LVEF, chamber volumes, E/e′ ratio, septal and lateral e′ velocities, tricuspid anular plane systolic excursion (TAPSE), and tricuspid S′), helping identify acute decompensation and biventricular involvement in fulminant presentations. It also allows detection of regional wall-motion abnormalities, which may appear patchy, non-coronary, or global, and reveals pericardial effusion—a finding present in a substantial subset of myocarditis and IMPS cases. Serial echocardiographic examinations are essential to document the temporal evolution of ventricular function and to assess improvement after initiation of immunosuppressive therapy. In the large international JACC CardioOncology 2025 cohort of 707 patients with ICI myocarditis, approximately one-third presented with an LVEF < 50% while two-thirds maintained preserved systolic function. Importantly, patients with reduced LVEF had markedly higher rates of shock, ventricular arrhythmias, mechanical circulatory support, and mortality [50].

In the context of a previously diagnosed or strongly suspected ICI myocarditis, GLS is much more accurate than LVEF in identifying who is at risk of MACE, especially if LVEF is still within the normal range. In the multicenter study by Awadalla et al. (JACC), 101 patients with ICI myocarditis were compared with 92 ICI-treated controls using centralized GLS analysis. At the time of myocarditis, GLS declined markedly to ~−14%, while it remained unchanged in controls. GLS was also a strong prognostic marker. About half of the myocarditis patients experienced MACE, and each 1% worsening in GLS nearly doubled the event risk, independently of LVEF. The predictive value was particularly pronounced in patients with preserved EF. A GLS threshold around −15/−16% effectively separated high-risk patients (≈60–70% MACE) from those at low risk (<10%). In contrast, baseline GLS was similar between groups (~−20%), indicating no predictive value before immunotherapy [51]. In the retrospective study by Quinaglia et al. [52], 75 patients with ICI myocarditis underwent core-lab strain analysis and were compared with ICI-treated controls without myocarditis. During the acute phase, both GCS and GRS were significantly impaired in myocarditis cases, not only relative to controls but also compared with their own pre-ICI values. Strain parameters (GLS, GCS, GRS) showed stronger prognostic performance than conventional markers. GCS and GRS demonstrated higher AUCs for predicting MACE than LVEF, troponin T, or age. Practical cut-offs—GCS around −18% and GRS around 40%—identified patients at markedly increased risk, with event rates exceeding 70%, compared with <10% in those above these thresholds [52].

When a full assessment of LV deformation is available (GLS, GCS, and GRS), risk stratification in ICI myocarditis becomes even more precise. Markedly impaired strain values identify patients at very high risk, supporting early ICU admission, a low threshold for mechanical circulatory support, and a more aggressive immunosuppressive strategy. Conversely, when strain parameters are relatively preserved—even in the presence of marked troponin elevation—the overall risk appears lower, which may justify a more measured intensity and duration of steroid therapy, although this remains based on expert consensus rather than definitive evidence.

On the contrary, we still do not have robust evidence to use serial GLS as routine screening in all patients undergoing ICI to “predict” who will develop myocarditis. In addition to the study by Awadalla et al., the Turkish study by Coskun [53], which prospectively followed patients treated with ICIs by measuring GLS in series to detect myocarditis or LV dysfunction early, did not show a significant role in the early prediction of myocarditis or cardiac dysfunction related to ICIs, and the authors emphasize the need for larger studies with longer follow-up [53].

The ESC 2025 guidelines on myocarditis and pericarditis recommend, in all patients with suspected myocarditis (including ICI-related forms), a comprehensive initial assessment that includes medical history, physical examination, ECG, biomarkers, and transthoracic echocardiography (TTE) as part of the basic procedure, both in the emergency department/hospitalization and in the outpatient setting. For ICI-induced myocarditis, the ESC 2025 guidelines emphasize the need for diagnostic triage within 24 h of suspected toxicity and immediate discontinuation of ICI and high-dose steroids but refer to the general guidelines for myocarditis imaging (i.e., TTE + CMR, EMB in high-risk cases). The ESC 2022 cardio-oncology guidelines suggest using strain mainly in high-risk patients (pre-existing heart disease, multiple cardiotoxicities, high cumulative doses) rather than as indiscriminate surveillance [38].

Another clinical use of TTE in immune-related myocarditis involves monitoring response to therapy. In Awadalla, in the few patients with follow-up TTE after steroids, GLS tended to improve (from ~−14% to ~−17%), albeit with small numbers and p not always significant.

With improvement in LVEF and strain, it is possible to gradually reduce steroids and immunosuppressants and begin discussing physical activity/rehabilitation. In cases of persistent LV dysfunction or significantly altered strain, however, immunosuppressive therapy should be maintained for longer or second-line therapy considered. It is also recommended to optimize therapy for heart failure and to be more cautious about re-challenging ICIs, a grey area, but imaging endpoints are fundamental to multidisciplinary decisions.

The ACC/ESC recommendations include an echo (with strain if possible) within 2–4 weeks of discharge in medium–high risk cases, and a new echo at 6 months, together with CMR, to determine recovery vs. progression to inflammatory cardiomyopathy [54].

Current limitations of echocardiography in ICI myocarditis include its modest sensitivity in early or mild disease, where LVEF is often normal and GLS may show only subtle, non-specific changes. GLS measurements also suffer from technical variability related to vendor differences, image quality, heart rate, and ectopy. No universally accepted strain cut-offs exist to define ICI myocarditis, as the proposed thresholds from Awadalla and Quinaglia are based on selected, retrospective cohorts. Finally, echocardiography cannot characterize myocardial tissue directly—edema, necrosis, and fibrosis remain the domain of CMR or EMB.

CMR: Recent evidence highlights important limitations and evolving opportunities for cardiac magnetic resonance (CMR) in the evaluation of immune checkpoint inhibitor (ICI)-related myocarditis, particularly in its subtle or “occult” phenotypes. In the largest multicenter cohort to date, Zhang et al. showed that conventional CMR criteria have modest sensitivity in ICI myocarditis, with late gadolinium enhancement (LGE) and T2 edema detectable in fewer than half of patients and even less frequently when left ventricular function is preserved; diagnostic yield increased substantially only when imaging was delayed beyond the early inflammatory window [40]. A systematic review by Li et al. further demonstrated that quantitative mapping techniques and strain imaging outperform traditional LGE- and T2-weighted sequences in detecting subclinical ICI injury: native T1/T2 values consistently rose in affected patients, whereas LGE and extracellular volume (ECV) often remained unchanged. Importantly, T2 elevation emerged as the only parameter associated with major adverse cardiovascular events, supporting the prognostic value of edema-sensitive mapping in this setting [55]. Reflecting these data, the 2024 ACC Expert Consensus Pathway underscores CMR—particularly T1/T2 mapping—as a pivotal test for noninvasive diagnosis of myocarditis, yet acknowledges that negative CMR does not exclude disease, especially when performed very early or in technically challenging patients. The document reaffirms the updated Lake Louise Criteria while emphasizing the need for high-quality mapping, adequate scan timing, and a low threshold for endomyocardial biopsy when suspicion persists [54]. Tissue mapping techniques, particularly native T1, are the primary imaging techniques in ICI-M. Native T1, associated with diffuse fibrosis, is the imaging marker with the highest concordance with EMB and has maintained significant prognostic value. An increase in native T1 can be correlated with both edema and fibrosis. T2 mapping assesses myocardial oedema [40].

Together, these findings position CMR as a cornerstone modality in ICI myocarditis, while clarifying its limitations and supporting an integrated diagnostic strategy that leverages biomarkers, mapping techniques, and selective biopsy to detect even the most muted inflammatory phenotypes.

EMB: Despite its limitations, EMB remains the gold standard for the definitive diagnosis of myocarditis. EMB should be considered in cases where the diagnosis is suspected but not non-invasively confirmed [56].

## 7. Focus on Emerging Biomarkers

The diagnosis and treatment of myocarditis are rapidly evolving, with the emergence of new biomarkers and techniques that enhance the understanding and management of this complex disease.

### 7.1. Standard Cardiac and Non-Cardiac Biomarkers

cTn represent the cornerstone of monitoring in ICI-associated myocarditis, with a sensitivity reaching 94% [57]. However, the specificity of cTnT can be compromised by the frequent concomitance of myositis, making cTnI a more accurate marker for specific myocardial injury [43]. Beyond its diagnostic utility, troponin plays a crucial prognostic role: cTnT levels exceeding 32 times the upper limit of normal within the first 72 h identify patients at extremely high risk of MACE, highlighting the need for early therapeutic escalation toward second-line immunosuppressive agents [43].

In addition to cTn, brain natriuretic peptide (BNP) and N-terminal pro-B-type natriuretic peptide (NT-proBNP) are also markers used to identify signs of myocardial injury and functional abnormalities [58,59]. The ratio between the decrease in absolute lymphocyte count (ALC) and the increase in neutrophil-to-lymphocyte ratio (NLR) has been associated with ICI-M. A greater decrease in ALC or an increase in NLR has been linked to subsequent MACE [60].

### 7.2. Novel Biomarkers: MicroRNA and Omics

Recent years have seen significant advancements in the identification and application of emerging biomarkers.

Free light chains (FLCs) have emerged as novel biomarkers of inflammation. A significant reduction in the FLCs κ/λ ratio has been observed in patients with myocarditis, independently correlated with prognosis, although this still needs to be validated in ICI-treated patients [61].

MicroRNAs are potential biomarkers for acute myocarditis. Serum exosomal levels of miR-155 and miR-320a have been proposed as non-invasive indicators for fulminant myocarditis. A multivariate logistic regression model combining miR-155 and miR-320a can accurately predict fulminant myocarditis with a significantly higher area under the ROC curve compared to cTn [62,63]. MiR-721, produced by TH17 cells, has been found elevated in the plasma of patients with myocarditis [63]. Transcriptomic analysis of circulating miRNAs revealed an upregulation of miR-4763-3p in patients with fulminant myocarditis, which normalizes after appropriate treatment [64]. However, these biomarkers need large-scale studies to be validated.

Next-generation sequencing (NGS) has revealed an increasing number of gene variants and mutations associated with the risk of heart disease, including dilated cardiomyopathy. Integrating these “omics” techniques, such as genomics, epigenomics, proteomics, and metabolomics, into existing diagnostic protocols could aid in the diagnosis and personalized treatment of ICI-M [65]. For example, comparative transcriptomic analysis of ICI-induced myocarditis has identified dysregulation of guanylate-binding proteins 5 and 6 [65].

Table 3 summarizes the advantages and limitations of non-invasive diagnostic methods.

## 8. Immunosuppressive Management and Rechallenge

The management of ICI myocarditis is multifactorial and requires a collaborative interdisciplinary approach involving oncologists, cardiologists, and intensive care unit staff.

### 8.1. First-Line Treatment

The first step in the treatment of all suspected cases of ICI-M is the immediate discontinuation of ICI therapy to prevent further toxicity [66]. Corticosteroids are the recommended first-line treatment for cardiovascular irAEs. Early intervention with corticosteroids is critical to reduce MACE, including mortality. The standard protocol involves initiating treatment with an intravenous (i.v.) bolus of methylprednisolone at a dosage of 500–1000 mg once daily for the first 3–5 days. After observing clinical improvement, such as a reduction in cTn greater than 50% from the peak level within 24–72 h and resolution of AV block or arrhythmias, a switch to oral prednisolone is recommended. The recommended initial oral dose is 1 mg/kg, up to a maximum of 80 mg/day [67].

### 8.2. Second-Line Therapy for Steroid-Refractory Cases

Up to 50% of patients may be refractory to corticosteroids [68]. In these cases, or in patients with fulminant/severe myocarditis, second-line immunosuppressive treatments are necessary. The decision on the optimal regimen must be made by a multidisciplinary team (MDT) in the absence of large-scale clinical trials. Second-line treatments include several drugs.

Mycophenolate mofetil (MMF) is frequently used to inhibit B- and T-cells, especially in cases unresponsive to corticosteroids, due to its established role in transplant rejection prevention and autoimmune disease management [69].

IVIG is employed to remove circulating antibodies via non-specific immunoadsorption. Although it has shown efficacy in several autoimmune diseases, its role in ICI-related myocarditis requires further validation [70,71].

Anti-thymocyte globulin (ATG) is an antibody preparation derived from the plasma of horses or rabbits that have been immunized with human thymocytes (T-cells). It works by recognizing and destroying T-lymphocytes (a type of white blood cell) in the circulation [72,73]. Alemtuzumab binds to CD52, triggering the destruction and profound depletion of these cells via immune mechanisms like antibody-dependent cellular cytotoxicity [74].

Abatacept is a fusion protein containing the extracellular domain of CTLA-4. Abatacept, combined with ruxolitinib, a JAK inhibitor, and screening for concomitant respiratory muscle insufficiency, has been associated with improved survival in cases of fulminant disease. Abatacept and ruxolitinib (a JAK1/JAK2 inhibitor) are considered emerging therapies [75].

Tocilizumab binds to both the soluble and membrane-bound forms of the IL-6 receptor. By blocking these receptors, it prevents IL-6 from binding and activating the cell, effectively shutting down the IL-6 signaling pathway and reducing systemic inflammation [76]. It is used for refractory severe ICI-M [76].

Infliximab is an anti-TNF-alfa that may be used as a rescue therapy when standard high-dose corticosteroids fail. However, the use of infliximab in an initial large-scale clinical trial (RENAISSANCE [77]) investigating high-dose infliximab for patients with moderate to severe chronic heart failure (NYHA Class III-IV) was stopped early because the drug appeared to increase the risk of death or hospitalization in these patients.

Plasmapheresis is recommended in combination with the agents mentioned above to rapidly eliminate circulating ICIs and inflammatory cytokines, especially in refractory or life-threatening cases [78].

### 8.3. ICI Rechallenge

irAEs are paradoxically associated with better anti-tumor responses, making ICIs a “double-edged sword”. The decision to resume ICI treatment after recovery from ICI-M is debated and must be approached with extreme caution due to the acute and potentially fatal nature of myocarditis [79].

Rechallenge is possible in selected patients, but a Multidisciplinary Team (MDT) discussion is strongly recommended, in line with the 2022 ESC guidelines on cardio-oncology [23]. The MDT evaluates the patient’s general health, the risk of irAE recurrence, and adapts the therapeutic strategy, optimizing both cardiovascular safety and oncological efficacy [23,80].

In patients who discontinued CTLA-4/PD-1 blockade due to severe irAEs, resuming anti-PD-1 therapy showed relatively high rates of recurrent or distinct toxicities, although manageable. However, specific data on rechallenge after high-grade ICI-M are scarce. The initial severity of myocarditis plays a significant role in the decision to rechallenge. Table 4 summarizes recommendations for ICI-M discontinuation and rechallenge.

## 9. Future Perspectives and Conclusions

Although myocarditis remains a rare complication of immune checkpoint inhibitors, its prevalence may be increasing, and the number of pauci-symptomatic cases with uncertain clinical evolution is also likely to rise. Timely and accurate diagnosis is essential not only to prevent major adverse cardiac events in the most severe cases, but also—perhaps even more importantly—to avoid depriving oncology patients of an effective therapeutic option when myocarditis is mild and non-progressive. Currently, diagnosis relies on a combination of clinical symptoms, biomarkers, ECG, echocardiography, and CMR. CMR, especially with T1 and T2 mapping techniques, plays a vital role in non-invasive diagnosis and risk stratification. However, CMR may have lower sensitivity in ICI-M compared to other forms of myocarditis, underscoring the importance of a multi-modal assessment and, if necessary, EMB.

First-line treatment involves immediate discontinuation of ICI and the use of high-dose corticosteroids. For refractory cases, second-line agents like MMF, Abatacept, and Ruxolitinib represent promising options that require further validation in large-scale clinical trials.

Regarding Abatacept, a phase 3 trial [81] is enrolling patients to evaluate whether adding Abatacept to corticosteroids reduces MACE. Therefore, a phase II trial [82], the Abatacept dose-finding phase II trial for ICI myocarditis (ACHLYS) trial, showed that the association of Abatacept and Ruxolitinib seems to significantly lower mortality rates from 60% to 3% in small, selected cohorts; however, definitive trial outcomes are still pending. Recently, a preclinical study [82] suggests that ICI-associated myocarditis is driven by the expansion of C-X-C motif chemokine receptor 6 (CXCR6)+ T-cells, identifying CXCR6 as a potential therapeutic target for this highly morbid condition.

Future perspectives focus on refining murine models to mimic human ICI-M better, understanding the pathogenic mechanisms, and identifying predictive biomarkers of toxicity and disease severity, thereby enabling more precise and targeted immunosuppressive regimens. Strategies are needed to balance the suppression of ICI-M with the maintenance of anti-tumor efficacy.

**Figure 2:**
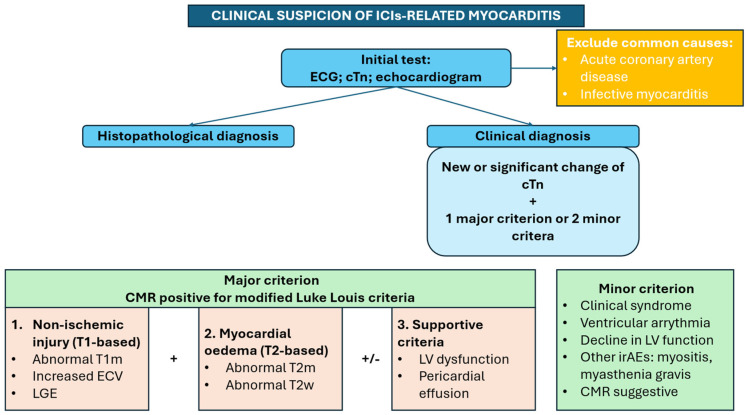
ECG: electrocardiogram; CMR: cardiac magnetic resonance; cTn: cardiac troponin; ICIs: immune checkpoints inhibitors; irAEs: immune-related adverse events; LGE: late gadolinium enhancement; LV: left ventricle; T2m: T2 mapping; T2w: T2 weight.

In conclusion, the management of ICI-M requires continuous, collaborative, and interdisciplinary effort. Figure 2 represents the diagnostic tool in case of clinical suspicion of ICI-related myocarditis.

## Figures and Tables

**Table 1:** Immune checkpoint inhibitors and their indications in several types of cancer.

Tumor Type	CTLA-4 (Global)	PD-1 (Global)	PD-L1 (Global)	PD-1 China-Only Agents
Melanoma	Ipilimumab; Ipilimumab + Nivolumab	Pembrolizumab; Nivolumab; Relatlimab + Nivolumab	–	Toripalimab; Camrelizumab
RCC	Ipilimumab + Nivolumab	Pembrolizumab; Nivolumab	Avelumab; Atezolizumab	–
CRC (MSI-H)	Ipilimumab + Nivolumab	Pembrolizumab; Nivolumab	–	Tislelizumab; Sintilimab
HCC	Tremelimumab + Durvalumab	Pembrolizumab; Nivolumab	Durvalumab; Atezolizumab + Bevacizumab	Camrelizumab; Sintilimab
NSCLC	Ipilimumab + Nivolumab; Tremelimumab + Durvalumab	Pembrolizumab; Nivolumab; Cemiplimab; Tislelizumab	Atezolizumab; Durvalumab	Toripalimab; Sintilimab; Camrelizumab; Tislelizumab
SCLC	–	–	Atezolizumab; Durvalumab	Camrelizumab
PM	Ipilimumab + Nivolumab	Nivolumab	–	–
HNSCC	–	Pembrolizumab; Nivolumab	–	Camrelizumab; Sintilimab
cHL	–	Pembrolizumab; Nivolumab	–	Sintilimab; Camrelizumab
PMBCL	–	Pembrolizumab	–	–
Urothelial carcinoma	–	Pembrolizumab; Nivolumab	Atezolizumab; Avelumab; Durvalumab	Toripalimab; Camrelizumab
Gastric/GEJ cancer	–	Pembrolizumab; Tislelizumab	–	Camrelizumab; Sintilimab
Esophageal SCC	–	Pembrolizumab; Nivolumab; Tislelizumab	–	Sintilimab; Camrelizumab; Tislelizumab
Cervical carcinoma	–	Pembrolizumab	–	Toripalimab; Camrelizumab
MCC	–	Pembrolizumab; Nivolumab	Avelumab	–
Endometrial carcinoma	–	Dostarlimab; Pembrolizumab	–	Camrelizumab
cSCC	–	Cemiplimab; Pembrolizumab	–	–
BCC	–	Cemiplimab	–	–
TNBC	–	Pembrolizumab	Atezolizumab	Camrelizumab; Toripalimab
BTC	–	Pembrolizumab	Durvalumab + Gemcitabine/Cisplatin	Camrelizumab
Nasopharyngeal carcinoma	–	Toripalimab; Tislelizumab	–	Toripalimab

BCC: Basal cell carcinoma; BTC: Biliary tract cancer; CTLA-4: cytotoxic T-lymphocyte antigen-4; cHL: Classical Hodgkin’s lymphoma; CRC: Colorectal cancer; cSCC: Cutaneous squamous cell carcinoma; GEJ: gastroesophageal junction; HCC: Hepatocellular carcinoma; HNSCC: Head and neck squamous cell carcinoma; MCC: Merkel cell carcinoma; MSI-H; microsatellite instability—high; NSCLC: Non-small cell lung cancer; PM: Pleural mesothelioma; PMBCL: Primary mediastinal large B-cell lymphoma; PD-1: programmed death 1; PD-L1: programmed death-ligand 1; RCC: Renal cell carcinoma; SCC: Squamous cell carcinoma; SCLC: Small cell lung cancer; TNBC: Triple-negative breast cancer.

**Table 2:** Classification of grades of myocarditis sec. common terminology criteria for adverse events (CTCAE), version 5.0, and European Society of Cardiology (ESC).

CTCAE Grade and Clinical Implications	ESC Classification and Clinical Implications
Grade 1 Myocarditis: N/A.	Low risk: Stable symptoms or oligosymptomatic.
Grade 2 Myocarditis: Symptoms with moderate activity or exertion.	Intermediate risk: New/progressive dyspnea; Non-sustained ventricular arrhythmias; Persistent release or relapsing troponin.
Grade 3 Myocarditis: Severe with symptoms at rest or with minimal activity or exertion; intervention indicated; new onset of symptoms.	High risk: Acute HF/cardiogenic shock; Dyspnea NYHA III–IV refractory to medical therapy; Cardiac arrest/syncope; Ventricular fibrillation/sustained ventricular tachycardia; High-level AV block.
Grade 4 Myocarditis: Life-threatening consequences: urgent intervention indicated, such as continuous intravenous therapy or mechanical hemodynamic support.	
Grade 5 Myocarditis: Death.	Death.

CTCAE: common terminology criteria for adverse events; ESC: European Society of Cardiology; NYHA: New York Heart Association.

**Table 3:** The advantages and limitations of non-invasive diagnostic methods.

Diagnostic Method	Pros/Advantages	Cons/Limitations
cTnT and cTnI	Highly sensitive (elevated in 94% of cases); cTnT is especially useful for monitoring and identifying risk (values ≥ 32× the upper limit within 72 h indicate high risk).	Elevated cTnT can reflect myositis rather than just the heart; lacks specificity for the exact cause of injury.
ECG	Essential first-line tool; abnormal in over 89% of cases.	Non-specific; ECG alterations are not predictive of the subsequent clinical course.
Standard Echocardiography	Crucial for initial hemodynamic triage and identifying acute decompensation or pericardial effusion.	Modest sensitivity for early screening; roughly 2/3 of patients have a normal LVEF at diagnosis.
Global Longitudinal Strain	Much more accurate than LVEF for identifying patients at risk of MACE, even when LVEF is normal.	Subject to technical variability; no universally accepted cut-offs yet.
Cardiac Magnetic Resonance	Cornerstone for tissue characterization; T1/T2 mapping can detect diffuse fibrosis and edema.	Modest sensitivity in very early stages; conventional criteria (LGE/T2 edema) are often negative when LVEF is preserved.
Novel Biomarkers	Potential for earlier diagnosis and more accurate prediction of fulminant cases than troponin.	Still largely in research phases; requires further validation in ICI-specific patient populations.

cTn: cardiac troponin; ECG: electrocardiogram; LGE: late gadolinium enhancement; LVEF: left ventricle ejection fraction; ICI: immune check points; MACE: major cardiovascular events.

**Table 4:** Recommendations for ICI-M discontinuation and rechallenge.

CTCAE Grade	ICI-Related Myocarditis	Recommendation for ICI Discontinuation	Recommendation for ICI Rechallenge
Grade 1	Mild; asymptomatic increase in cardiac biomarkers or mild fatigue.	Treatment suspension/hold: although typically used for managing grades 2–4, for mild cases, cessation or continuation with close monitoring may be clinically determined.	Rechallenge may be considered if symptoms and/or lab values resolve or return to ≤grade 1.
Grade 2	Symptoms present with moderate activity or exertion.	Hold recommended: the ICI therapy should generally be suspended immediately upon suspicion of myocarditis.	Rechallenge is sometimes possible if symptoms and/or lab values return to ≤grade 1.
Grade 3	Severe: symptoms at rest or with minimal activity or exertion; intervention indicated; new onset of symptoms. High risk of recurrent toxicity.	Permanent discontinuation recommended.	Generally not recommended: rechallenge is usually discouraged for grade 3 toxicity (except for endocrinopathies managed by replacement therapy, per broader irAE guidelines). Requires extreme caution and MDT discussion.
Grade 4	Life-threatening consequences: urgent intervention indicated.	Permanent discontinuation recommended.	Generally not recommended: rechallenge is strongly contraindicated for grade 4 toxicities (except highly controlled endocrine irAEs). Requires MDT discussion.

CTCAE: common terminology criteria for adverse events; ICI: immune checkpoint inhibitors; irAEs: immune-related adverse events; MDT: multidisciplinary teams.

## Data Availability

No new data were created or analyzed in this study.
